# Expectations of orthodontic treatment among 7–12 year-old children – a cross sectional study

**DOI:** 10.2340/aos.v84.43910

**Published:** 2025-06-18

**Authors:** Anne-Maria Aulu, Tuija Pikkusaari, David Rice, Heidi Arponen

**Affiliations:** a Wellbeing Services County of Vantaa and Kerava, Vantaa, Finland; b Department of Oral and Maxillofacial Diseases, University of Helsinki, Helsinki, Finland; c Head and Neck Center, Helsinki University Hospital, Helsinki, Finland

**Keywords:** orthodontics, treatment expectations, children, treatment need, patient reported outcomes

## Abstract

**Objective:**

The primary aim of this study was to investigate participants’ orthodontic treatment expectations and to assess participants’ perceptions of their own orthodontic treatment need. A secondary aim was to compare participants’ self-perceived treatment need to an orthodontist’s opinion.

**Material and methods:**

In this cross-sectional study, a total of 98 participants, aged 7–12 years, completed a modified Sayers questionnaire before an orthodontic screening in a public health center in Finland. After completing the questionnaire, the participants underwent a clinical examination of the occlusion by an orthodontist specialist. A Spearman’s rank correlation was conducted to evaluate the relationship between the questionnaire answers. The chi square statistic was applied to assess association between categorical variables.

**Results:**

From the initial orthodontic screening, a sample of 98, 94 participants were included in further analysis. Of them, 89% expressed a self-perceived need for orthodontic treatment. Orthodontic treatment need was determined by an orthodontist for 66% of the participants based on a severe malocclusion. Gender, age, or family history of orthodontic treatment were not associated with subjective treatment need. The main expectations for orthodontic treatment result were to straighten the teeth, to get a better smile, and to avoid problems in the future.

**Conclusions:**

The study found that patients’ expectations of treatment did not fully align with reality. More information for patients and their guardians is needed in advance to avoid unrealistic expectations before the orthodontic treatment.

## Introduction

In Finland, the majority of children’s orthodontic treatment is performed in the public healthcare system. Treatment in the public healthcare is supported by the government, and the treatment is free of charge for individuals under the age of 18 years. Treatment is offered to children, with a malocclusion that fulfills predetermined criteria. The focus is in the functional problems. If a child has an esthetic problem or only a mild malocclusion, it is not treated in the public healthcare [1]. Generally, the occlusion is classified using a 10-grade scale created by Heikinheimo [2]. Heikinheimo’s 10-grade scale is based on the Grainger’s Treatment Priority Index and orthodontic treatment is offered to all children with a grade of eight or above in Finnish public health care [3, 4]. Malocclusions which fulfill the criteria include cleft lip and palate, other severe developmental disorders of the head and jaws, impacted canines and upper incisors, severe distal occlusion, severe progenia, severe openbite, severe deepbite, severe hypodontia, anterior and posterior crossbites with functional problem, severe crowding and scissorsbite [2]. Grading of the malocclusion is done usually by orthodontists who are trained to apply the 10-grade scale but not calibrated. The typical age for orthodontic screening and grading of the malocclusion in Finland is between 6 and 12 years.

In the year 2022, 28% of 12 years old children (31% of females, 26% of males) were in orthodontic treatment in the public healthcare in Finland. The percentage of the treated 12-year olds in the Vantaa and Kerava region, where the present study was conducted, was similarly 28% in the year 2022 (32% of females, 24% of males) [5]. The mean age for starting orthodontic treatment in Finnish health centers varies from 7.8 to 11.7 years of age [1, 6].

Orthodontists frequently encounter situations where a patient or family desires treatment, yet it is not offered due to the patient’s malocclusion not meeting the predetermined criteria of public healthcare. Conversely, there are instances where treatment is recommended, but the patient or family declines to proceed. Previous studies have investigated the opinions of patients and their guardians regarding the need for orthodontic treatment [7–12]. However, Livas et al. concluded in their systematic review, that assumptions rather than reliable conclusions can be made about the association between professional and self-assessment treatment need [8]. A systematic review by Theodoridou et al. has stated that the need for orthodontic treatment should be prioritized considering not only normative need but patients’ and parents’ perspectives in terms of oral health-related quality of life (OHRQoL) of patients [13]. The findings of this review study highlighted an association between the need for orthodontic treatment and OHRQoL. From the results of this study it could be seen that each person perceives his/her quality of life differently, and the OHRQoL may be affected by malocclusion. Additionally, OHRQoL is not only about function. The study highlights that the psychosocial background of each patient should be taken into account before providing treatment. Thus, the goal of treatment should be to promote oral health and OHRQoL in terms of functional as well as social and emotional aspects [13].

Divergent perspectives among patients, their guardians and orthodontists have been demonstrated regarding expectations, treatment necessity and objectives of orthodontic care [14–17]. Previous studies have concluded that patients and parents need thorough information before the starting of treatment to avoid unrealistic expectations [15]. It has also been stated, that patients tend to be happier post-treatment when their expectations align closely with the actual outcomes, although the evidence concerning this is weak [18, 19].

Taken together, while there is data that patients’ and parents’ perspectives may differ from that of orthodontists’, relatively little has been published comparing the opinion of the patient and orthodontist with regard to treatment need, treatment expectations and relating these to malocclusion type. Additionally, recent literature search by Liu et al. revealed that orthodontic research outputs are mostly clinician-focused and more efforts should be focused on conducting studies that include patient-reported outcomes [20].

The primary aim of this study was to evaluate participants’ expectations on orthodontic treatment prior to screening of the occlusion and their opinion of their own orthodontic treatment need. A secondary aim was to compare participants’ self-perceived treatment need to an orthodontist’s opinion.

## Materials and methods

The Standards for Reporting Qualitative Research (SRQR) guidelines by O’Brien at al. were followed in this study [21]. This study was approved by the ethical committee of HUS – Helsinki University Hospital (HUS/1483/2021 and VD/7227-/13.00.00/2021), and a research permission was granted by Vantaa and Kerava Wellbeing services county (HUS/1483/2021 and VD/7227/13.00.00/2021).

### Participants

The inclusion criteria were as follows: (1) patients aged 7–12 years; (2) patients who have not previously undergone orthodontic treatment; and (3) participants who could communicate and read in Finnish, Swedish or English. Participation in this study was voluntary, and patients, and guardians provided written informed consent. The participants were not representative of the general population but rather a random sample of children pre-selected by general practitioners for an orthodontic screening based on a suspected malocclusion.

### Study protocol

In this cross-sectional study, participants and one of their guardians were asked to complete a psychometrically validated questionnaire [22] before a screening appointment at an orthodontist office at the Wellbeing services county of Vantaa and Kerava healthcare center in Finland. The questionnaire used has been tested previously for validity and reliability [22] and has been used in different populations [15, 16, 22–24]. The original questionnaire was translated from English into Finnish and Swedish by two individuals for the present study. Subsequently, the translated versions were compared against the original questionnaire by a third trilingual investigator, native in English language. The questionnaire was modified by removing the original questions 1a–f (Appendix 1). These questions didn’t concern the present study. Three questions were added; question number 1 investigates participant’s own opinion concerning the orthodontic treatment need, question number 2 investigates family’s previous experience of orthodontic treatment, and question number 11h investigates participant’s expectations of orthodontic treatment (Appendix 1). The questionnaire used a visual analog scale response code, marked at 1-mm intervals, ranging from 0 (‘extremely unlikely’) to 100 (‘extremely likely’); and 2 questions (9 and 10) used categorical response codes. The millimeter distance between the mark on the visual analog scale (participant’s response) and left-hand side of the scale (‘0’) was measured to assess the scores for questions 3a–e, 4–8 and 11a–h; and questions 9 and 10 had 11 and 10 response options, respectively. Question numbers 1 and 2 had three answering choices (yes, no, maybe). This questionnaire measures orthodontic treatment expectations in regard to the treatment need, the family’s previous experience of orthodontic treatment, the type of treatment, expected experiences during orthodontic treatment, treatment duration, frequency of visits, and benefits of orthodontic treatment.

The children, accompanied by their guardians, jointly completed the questionnaire in the waiting room prior to their orthodontic screening appointment. While the children were asked to provide their own opinions on the questions, guardians assisted in filling out the questionnaire. The study group contained patients aged 7–12 years, and it was known beforehand that participants need the help of guardian to fill out the questionnaire. This age group was chosen since this is the typical group having orthodontic screening in Finland [4, 6]. In other words, this is the real life situation. If a participant answered ‘no’ to question number 1 (no self-perceived need for orthodontic treatment), she/he was instructed not to answer to the subsequent questions. Following the completion of the questionnaire, the children received their orthodontic consultation, which was conducted by an orthodontist specialist (A.A or H.A). The orthodontists (A.A and H.A) who did the screening, were not calibrated. During the consultation the following information was recorded: symmetry of face, profile, developmental stage of dentition, canine palpation, angle-classification of molars and canines, overjet, overbite, crossbite, scissorsbite, traumatic deepbite, openbite, crowding, diastema (Table 1). Crowding was classified as mild, moderate or severe. The malocclusion severity was scored according to 10-grade scale [2]. In addition, the potential treatment options were recorded, indicating whether treatment was provided or not. Furthermore, patients classified as ‘treatment provided’ were assigned an ICD-10 diagnosis code (International Statistical Classification of Diseases and Related Health Problems) [25].

**Table 1:** Occlusion traits recorded in orthodontic consultation.

Trait recorded	Categorisation
Symmetry of face	yes/no
Profile	convex/concave/straight/harmonic
Developmental stage of dentition	primary dentition/mixed dentition/permanent dentition
Canine palpation	labial/lingual/no palpation/erupted
Angle-classification of molars	AI/AII/AIII
Angle-classification of canines	AI/AII/AIII
Overjet	mm
Overbite	mm
Crossbite	yes/no, anterior/posterior, functional/no functional
Scissorsbite	yes/no
Traumatic deepbite	yes/no
Openbite	yes/no, anterior/posterior
Crowding	no/miId/moderatc/severe
Diastema	mm

mm: millimetre.

### Sample size

Sample size estimate was based on previous studies applying the Sayers questionnaire on study populations varying between 50 and 110 [14–16, 24]. A sample size of 134 subjects [67 patients and one parent each (*n* = 67)] has been shown to achieve a 80% power to detect a 7-mm difference, with a standard deviation (SD) of 20, between patients’ and parents’ responses, with significance level set at 0.05 [14]. In the present study the target for the sample size was 80, that is to say 80 patients and one guardian each (*n* = 80).

### Statistics

Intra-class correlation coefficient (ICC) two-way mixed-effects model was used to evaluate the reliability among the questionnaire result interpretation, each model independently analyzed. Normality of the continuous data was tested with Kolmogorov-Smirnov test of normality. A Spearman’s rank correlation was conducted to evaluate the relationship between the questionnaire answers. The chi square statistic was applied to assess association between categorical variables, including association between subjective and objective treatment need.

The consultation records, the questionnaires and all the responses were analyzed by one examiner (A.A). After study completion, the same examiner (A.A) re-measured 20 randomly selected questionnaires to evaluate intra-observer reliability.

Some data was missing at random. All the participants did not complete the whole questionnaire. To increase reliability of the analysis imputation was not conducted, instead pairwise deletion method was applied. Available data was analyzed for each analysis separately, ignoring missing values in other variables.

## Results

### Study participants

**Figure 1:**
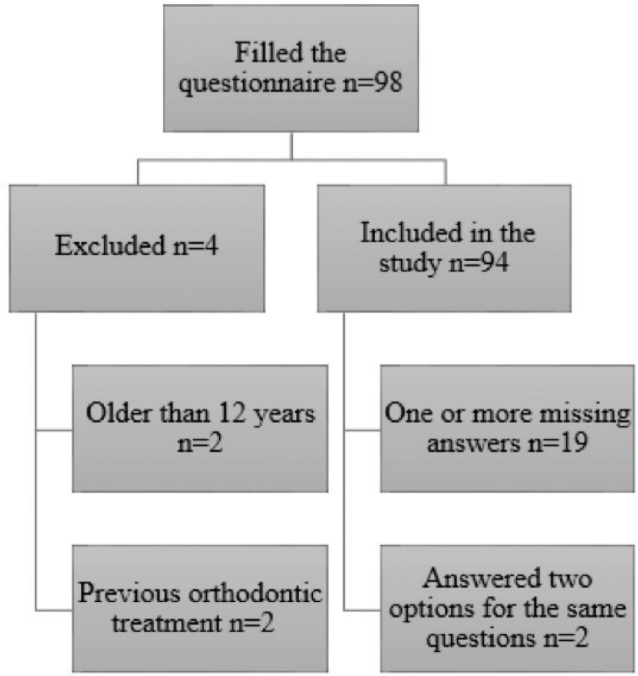
Flow chart.

A total of 98 individuals completed the questionnaire between September 2021 and September 2023. Two patients were excluded from the study since they were older than 12 years. Two additional patients were excluded from the study since they had previous orthodontic treatment. The final sample size was 94 (Figure 1).

The mean age of the participants was 9.6 (SD 1.4) years. The median age of the participants was 10 years. The youngest participant was 7 and oldest was 12 years old. Of the participants, 48 were girls and 46 boys. In total, 64 (58 yes, 6 maybe) participants reported a family history of orthodontic treatment (their parents and/or siblings underwent orthodontic treatment in the past). Of the participants, 19 did not complete the whole questionnaire, and had one or more missing answers. Two participants answered two options for the same question (question number 10).

A high degree of reliability was found between all the questionnaire result interpretations measurements. The ICC measure ranged between 0.724 (one variable, Q3, (*F* (18.18) = 6.247, *p* < 0.001)) and 1.0 indicating perfect agreement (19 variables). The questionnaire responses deviated statistically significantly from normal distribution (*p* < 0.001).

### Self-perceived need for orthodontic treatment and treatment need assessed by an orthodontist

**Figure 2:**
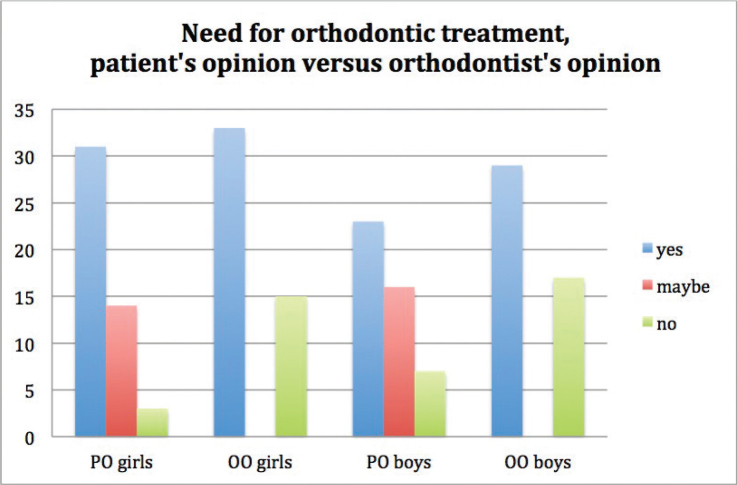
PO girls = patient’s opinion girls, OO girls = orthodontist’s opinion girls, PO boys = patient’s opinion boys, OO boys = orthodontist’s opinion boys. 84 participants out of 94 had self-perceived need for orthodontic treatment (yes, girls 31, boys 23; maybe, girls 14, boys 16). 10 individuals did not perceive the need for orthodontic treatment (girls 3, boys 7). Orthodontic treatment need was assessed by an orthodontist for 62 patients (girls 33, boys 29). 32 patients did not have orthodontic treatment need determined by an orthodontist (girls 15, boys 17).

In total, 84 participants out of 94 had self-perceived need for orthodontic treatment (yes, girls 31, boys 23; maybe, girls 14, boys 16). Ten participants did not perceive the need for orthodontic treatment (girls 3, boys 7) (Figure 2). Orthodontic treatment need was determined by an orthodontist for 62 patients (girls 33, boys 29). Thirty-two patients did not have orthodontic treatment need determined by an orthodontist (girls 15, boys 17) (Figure 2).

A self-perceived need for orthodontic treatment was reported by 27 patients, who were eventually not offered treatment. (Table 2) The self-perceived treatment need was not associated with the treatment need deemed by an orthodontist (*p* = 0.522). The 10 patients who did not perceive the need for orthodontic treatment are classified in Table 3. Among the participants who had objective need for orthodontic treatment assessed by an orthodontist, the most common ICD-10 diagnosis codes were K07.30 crowding, K07.23 deepbite and K07.22 overjet. (Table 4) Boys were equally likely to be deemed in need of treatment as girls (*χ*^2^ (1) = 0.341, *p* = 0.664). Gender or previous orthodontic treatment history of family members was not associated with subjective treatment need assessed by the participant/guardian (*χ*^2^ (2) = 2.877, *p* =0.237 and *χ*^2^ (1) = 2.158, *p* = 0.142 respectively). However, boys were more likely to anticipate getting some type of orthodontic brace than girls (*r*_s_ (78) = 250, *p* = 0.027). Age of the respondent was not associated with the subjective treatment need (*r*_s_ (94) = –0.028, *p* = 0.791). Younger participants were admitted to treatment more often than the older ones as expressed by a negative correlation between increasing age and deemed treatment need (*r*_s_ = –0.336, *p* = 0.001).

**Table 2:** Individuals (*n* = 27) who had self-perceived need for orthodontic treatment but the treatment was not offered.

Patient	Type of malocclusion
1	Mild crowding, crossbite on side but no slide, overjet 3, overbite 3
2	Mild crowding, deepbite (not traumatic), overjet 5,5, overbite 4
3	Mild crowding, one tooth in crossbite in front but no slide, overjet 2, overbite 2,5
4	No crowding, crossbite on side but no slide, overjet 2, overbite 2
5	Mild crowding, overjet 4,5, overbite 3,5
6	No crowding, crossbite on side but no slide, overjet 3, ovorbite 4
7	No crowding, overjet 2,5, overbite 3
8	No crowding, teeth 15, 25, 35, 45 missing
9	Moderate crowding, overjet 4, overbite 4
10	Moderate crowding, teeth 35, 45 missing, crossbite on side but no slide
11	Moderate crowding, overjet 2,5, overbite 2
12	No crowding, overjet 6, overbite 2
13	No crowding, deepbite (not traumatic), overjet 1,5, overbite 5,5
14	Moderate crowding, deepbite (not traumatic), overjet 7, overbite 6
15	Moderate crowding, overjet 6, overbite 2
16	Mild crowding, overjet 3,5, overbite 3,5
17	Diastema, overjet 4,5, overbite 5,5
18	No crowding, deepbite (not traumatic), overjet 7, overbite 5
19	Moderate crowding, overjet 5, overbite 3
20	No crowding, crossbite on side but no slide, overjet 4, overbite 4
21	No crowding, deepbite (not traumatic), overjet 6, overbite 5
22	Mild crowding, overjet 3,5, overbite 4
23	Mild crowding, deepbite (not traumatic), overjet 4, overbite 5,5
24	Diastema, overjet 6, overbite 4,5
25	Medial diastema 1,5mm, mild crowding, overjet 3, overbite 4
26	No crowding, deepbite (not traumatic), overjet 4,5, overbite 3,5
27	Mild crowding, deepbite (not traumatic), overjet 4,5, overbite 3,5

mm: millimetre.

**Table 3:** Classification of 10 individuals who did not perceive the need for orthodontic treatment.

Patient	Type of malocclusion	Treatment offered
1	Diastema, openbite in front, crossbite in front, overjet -2, overbite -3, prognatic mandible	yes
2	No crowding, medial diastema 1,5mm, overjet 2,5, overbite 1,5	no
3	No crowding, overjet 2,5, overbite 1,5	no
4	No crowding, deepbite (not traumatic), overjet 4,5, overbite 6	no
5	Moderate crowding, deepbite (not traumatic), overjet 3, overbite 5	no
6	Diastema, openbite in front, overjet 4, overbite -0,5	no
7	Medial diastema 3mm, traumatic deepbite, overjet 6, overbite 4, scissorsbite	yes
8	Diastema, medial diastema 3mm, overjet 9, overbite 4	yes
9	Medial diastema 2mm, diastema, severe crowding, impacted canine, overjet 4, overbite 2,5	yes
10	Crossbite on side, non-stable bite, overjet 1,5, overbite 1,5	yes

**Table 4:** ICD-10 diagnosis codes among the participants who had objective need for orthodontic treatment assessed by an orthodontist.

ICD-10 diagnosis codes	Number of patients
K07.30 crowding	27
K07.23 deepbite	25
K07.22 overjet	18
K07.25 crossbite lateral	10
K07.25 crossbite anterior	9
K07.27 scissorsbite	6
K01.12 impacted maxillary canine	6
K00.00 hypodontia/oligodontia	4
K07.33 diastema	4
K07.20 disto-occlusion	3
K07.24 openbite	2
K07.11 mandibular prognathism	1

One participant can have one or more diagnosis code.

### Treatment expectations

The results of participants’/families’ orthodontic treatment expectations are presented in Table 5. Statistically significant positive correlations between questionnaire answers (*p* ≤ 0.05*, *p* ≤ 0.001**) are presented in Table 6.

**Table 5:** The results of participants’ orthodontic treatment expectations on a scale from 0 to 10, where 0 indicates Extremely unlikely and 10 signifies Extremely likely.

	mean	standard deviation answers	*n*
**3) What type of orthodontic treatment do you (/your child) expect?**			
a) Braces, don’t know what type?	5.80	2.96	78
b) Train track braces?	3.82	2.41	74
c) Teeth removal?	1.05	1.67	77
d) Head brace or some other removable appliance placed into the mouth by yourself?	2.99	3.30	73
e) Jaw surgery?	0.50	1.68	78
**4) Do you think orthodontic treatment will give you any problems?**	1.95	2.63	78
**5) Do you think orthodontic treatment will be painful?**	3.60	2.86	79
**6) Do you think orthodontic treatment will produce problems with eating?**	4.88	2.89	79
**7) Do you expect orthodontic treatment to restrict what you (/ your child) can eat or drink?**	4.72	3.49	79
**8) How do you think people will react to you (/your child) wearing a brace?**	6.13	3.20	78
**11) Do you expect orthodontic treatment to:**			
a) Straighten your teeth?	8.73	2.26	77
b) Produce a better smile?	5.56	3.39	78
c) Make it easier to eat?	3.13	3.74	77
d) Make it easier to speak?	2.41	3.28	77
e) Make it easier to keep my teeth clean?	5.39	3.70	78
f) Improve my chances of a good career?	2.30	3.31	77
g) Give you confidence socially?	3.48	3.68	78
h) Problems will be avoided in the future?	7.13	3.48	79

On question number 8, 0 indicates negative reaction and 10 indicates positive reaction. Participants who answered ‘no’ to question number 1 (no self-perceived need for orthodontic treatment) were instructed not to answer to questions 3a–11h.

**Table 6:** Statistically significant positive correlations between questionnaire answers (*p* ≤ 0.05*, *p* ≤ 0.001**).

Question	SPN	FHO	3a	3b	3c	3d	3e	4	5	6	7	8	11a	11b	11c	11d	11e	11f	11g	11h
SPN										0.221*										
FHO												0.334*								
3a											0.308*		0.293*						0.323*	0.317*
3b																				
3c											0.233*		0.297*						0.242*	
3d											0.243*								0.341*	0.247*
3e															0.243*				0.327*	0.225*
4										0.362**										
5										0.598**										
6	0.221*							0.362**	0.598**		0.554**									
7			0.308*		0.233*	0.243*				0.554**										
8		0.334*																		
11a			0.293*		0.297*									0.237*			0.263*		0.340*	0.428**
11b													0.237*		0.274*	0.291*	0.435**		0.407**	0.333*
11c							0.243*							0.274*						
11d														0.291*						
11e													0.263*	0.435**						
11f																				
11g			0.323*		0.242*	0.341*	0.327*						0.340*	0.407**						0.328*
11h			0.317*			0.247*	0.225*						0.428**	0.333*					0.328*	

A Spearman’s rank correlation was conducted to evaluate the relationship between the questionnaire answers. SPN: self perceived need for orthodontic treatment; FHO: family history of orthodontics.

Earlier orthodontic treatment history in the family correlated positively with the expectation of how people will react to wearing braces (*r*_s_ (76) = 0.334, *p* = 0.030). Expectations of getting some kind of treatment correlated positively with positive expectations of the treatment results. Participants who expected to wear braces, expected orthodontic treatment to straighten teeth (*r*_s_ (75) = 0.293, *p* = 0.011), give social confidence (*r*_s_ (76) = 0.323, *p* = 0.004), and to help avoid problems in the future (*r*_s_ (77) = 0.317, *p* = 0.005). Expectation of teeth removal positively correlated with the expectations of teeth straightening and gaining greater social confidence (*r*_s_ (74) = 0.297, *p* = 0.010), *r*_s_ (75) = 0.242, *p* = 0.037). Additionally, expectation of being treated with removable appliance positively correlated with the expectations of gaining greater social confidence and avoiding problems in the future (*r*_s_ (71) = 0.341, *p* = 0.004, *r*_s_ (72) = 0.247, *p* = 0.037). Expectation of jaw surgery positively correlated with the expectations of avoiding problems in the future, gaining greater social confidence and eating being easier after orthodontic treatment (*r*_s_ (77) = 0.225, *p* = 0.049), *r*_s_ (76) = 0.327, *p* = 0.004), *r*_s_ (75) = 0.243, *p* = 0.036), respectively).

Participants/families had expectations concerning the problems during the orthodontic treatment. The main concerns were that orthodontic treatment would produce problems with eating (mean likelihood score 4.88 out of 10) and having eating and drinking restrictions during the treatment (mean likelihood score 4.72 out of 10). The expectation of wearing braces correlated positively with the expectation of having restrictions with eating and drinking (*r*_s_ (77) = 0.308, *p* = 0.007). The expectation of teeth removal positively correlated with the expectation of having restrictions with eating and drinking (*r*_s_ (77) = 0.233, *p* = 0.041). The expectation of getting removable appliance positively correlated with the expectation of having restrictions with eating and drinking (*r*_s_ (73) = 0.243, *p* = 0.038)). The expectation of having problems with eating positively correlated with the self-perceived need for orthodontic treatment and with the expectations of getting problems, treatment being painful and having restrictions with eating and drinking (*r*_s_ (79) = 0.221, *p* = 0.050); *r*_s_ (78) = 0.362, *p* = 0.001); *r*_s_ (79) = 0.598, *p* = 0.000), *r*_s_ (78) = 0.554, *p* < 0.001).

The main positive expectations for treatment were to straighten teeth (mean likelihood score 8.73 out of 10), produce a better smile (mean likelihood score 5.56 out of 10) and avoiding problems in the future (mean likelihood score 7.13 out of 10). Expectation of orthodontic treatment to straighten teeth positively correlated with the expectations of getting better smile, make it easier to keep teeth clean, gaining greater social confidence and avoiding problems in the future (*r*_s_ (77) = 0.237, *p* = 0.038), *r*_s_ (76) = 0.263, *p* = 0.022), *r*_s_ (77) = 0.340, *p* = 0.002), *r*_s_ (77) = 0.428, *p* = 0.000). Expectation of getting a better smile positively correlated with the expectations of orthodontic treatment making eating, speaking or cleaning teeth easier, gaining greater social confidence, and avoiding problems in the future (*r*_s_ (76) = 0.274, *p* = 0.017), *r*_s_ (76) = 0.291, *p* = 0.011), *r*_s_ (77) = 0.435, *p* = 0.000), *r*_s_ (77) = 0.407, *p* = 0.000), *r*_s_ (78) = 0.333, *p* = 0.003). Expectation of gaining greater social confidence positively correlated with the expectation of avoiding problems in the future (*r*_s_ (78) = 0.328, *p* = 0.003).

**Figure 3:**
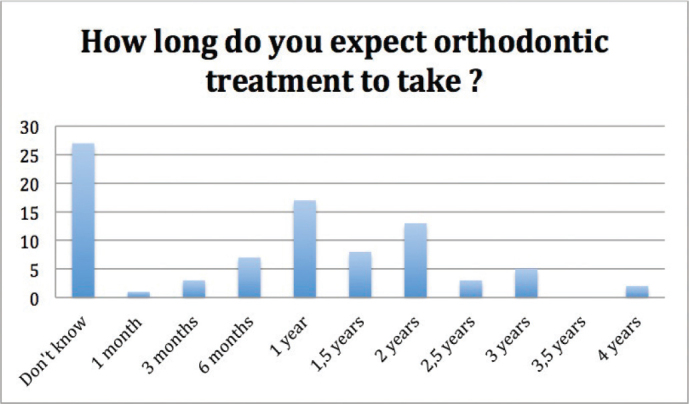
Expectations regarding the duration of orthodontic treatment. Three individuals did not answer to this question (*n* = 81).

**Figure 4:**
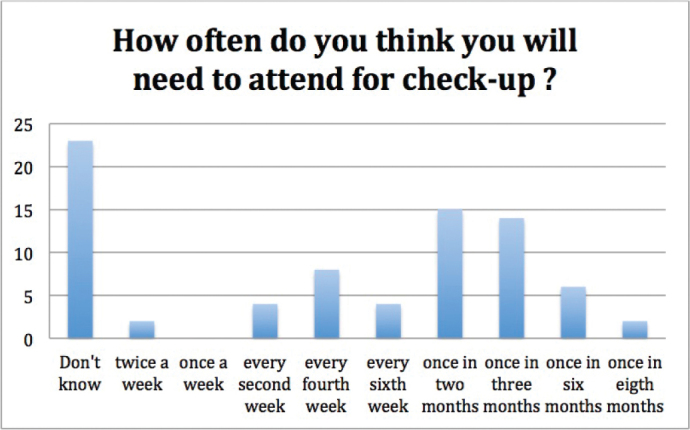
Expectations regarding the frequency of appointments. Two individuals answered two options for the same question; additionally five participants did not answer to this question (*n* = 76).

Expectations regarding the duration of orthodontic treatment (question 9) (Figure 3) and frequency of appointments (question 10) (Figure 4) did not correlate significantly with gender, age, or family history of orthodontic treatment (*p* = 0.244, *p* = 0.389, *p* = 0.866, *p* = 0.480, *p* = 0.784, and *p* = 0.848 respectively). Regarding the duration of orthodontic treatment, 27 participants answered ‘Don’t know’, and with respect to the frequency of appointments, 23 participants answered ‘Don’t know’.

## Discussion

In the present study, 7–12 year-olds children’s opinion of their own orthodontic treatment need was examined and compared to an orthodontist’s opinion in a publicly funded clinic. Additionally, participants’ orthodontic treatment expectations were investigated before orthodontic screening.

### Self-perceived need for orthodontic treatment and treatment need assessed by an orthodontist

In our selected sample, orthodontic treatment need was determined by an orthodontist for 66% of the participants. Previous data show that 28.3% of 12 years old children were in orthodontic treatment in the public healthcare in Finland in year 2022 [5]. The percentage is higher in our study as the participants were not representative of the general population but rather a coincidental sample of children pre-selected by general practitioners for an orthodontic screening based on a suspected malocclusion.

We found that 57% and 32% of the participants in the study had or evaluated that they would have a subjective need for orthodontic treatment, respectively. Only 11% of the participants did not consider they had a need for orthodontic treatment. Thus, the subjective orthodontic treatment need was higher than the severe treatment need defined according to the 10-grade scale by the orthodontist. However, only 57% of participants were certain of their orthodontic treatment need, 32% were hesitant and answered ‘maybe’. The sample age group of 7–12 years is the mixed dentition phase and hence most of the parents and children will feel the need for orthodontic treatment while dentition develops. In the future, information could be delivered for example through TV/radio/social media, that during mixed dentition families do not need to stress unless there are certain specific features that their kids are showing in their bite, such as open bite, cross bite, large overjet et cetera.

In a former study it has been concluded that girls experience more need for treatment compared to boys [7, 8, 26] In the present study gender was not associated with the subjective treatment need. Girls had more self-perceived need for orthodontic treatment but the difference did not reach statistical significance. It has also been concluded that older children experience more need for treatment compared to younger children [8]. In the present study, age of the respondent was not associated with the subjective treatment need but interestingly younger patients were selected to treatment more often than the older ones.

In the present study, many participants who were not offered but had a self-perceived need for orthodontic treatment, had increased overbite with no trauma, anterior crowding, large overjets and anterior tooth rotations. Earlier it has been stated that so called esthetic malocclusions increase the experience of orthodontic treatment need by patients. Such malocclusions are crowding in front, big overjet and rotations in front [8].

### Treatment expectations

Our study was in line with the previous studies about the patients’ expectations of orthodontic treatment. Patients expect orthodontic treatment to help straighten the teeth, having a better smile and gaining greater social confidence [14, 15]. In the present study, orthodontic treatment was also thought to prevent problems in the future. Functional aspects, such as improvements in speech and eating with orthodontic treatment, were not emphasized in the present study. Previously, it has also been stated that for children who experience bullying, ‘ugly teeth’ might be a major motivating factor to seek orthodontic treatment with the expectation that orthodontic treatment could prevent them from experiencing further bullying [27]. This former finding also highlights the importance of aesthetics for patients.

In the present study, the main concerns for orthodontic treatment were having problems with eating and having eating and drinking restrictions. However, the scale ranged from 0 to 10 and the mean response for problems with eating was only 4.88 and for eating and drinking restrictions 4.72. In general, participants had quite positive attitude towards the possible orthodontic treatment. It has been noticed earlier that patients and their parents do not expect orthodontic treatment to cause discomfort and difficulty in eating or drinking [15]. It is also interesting that in the present study earlier family history of orthodontic treatment correlated positively with the expectation of how people will react to patient wearing braces. However, patient’s attitude towards orthodontic treatment has been shown to be unpredicting of co-operation of a patient during the treatment [28].

Participants did not often know how long their orthodontic treatment could take or how often there would be treatment appointments: 33% and 30% patients were unaware of the duration of orthodontic treatment, and frequency of appointments, respectively. It is understandable, that it is difficult for the patient to assess the duration of their ortho-dontic treatment, as duration can vary a lot [1]. In a former study it has been stated that most patients and guardians underestimate the duration of orthodontic treatment and the number of visits needed [15].

### Strengths and limitations

In our study, the estimated necessary sample size was achieved. The sample size target was limited to 80 based on previous studies, and bearing in mind that this study was done during everyday health center work. In one previous study done on psychological impact of orthodontic treatment on quality of life, the sample size was larger [29]. As an advantage, selection bias is relatively low in the present study. This study was similar to a population screening, although it suffered from some selection bias, as the children were preselected by general practitioners based on suspected malocclusion. Also, all patients attending orthodontic screening could not be offered the possibility of attending the study because this study was committed during everyday health center work, and some preselection of the patients did happen when the comprehension and language skills of children and guardians was shortly assessed when offering the possibility of attending the study. Some respondents may have answered the questionnaire in Finnish, even though it may not have been their native language. Although the original questionnaire is validated, the modified and translated questionnaire we used in this study is not. The original questionnaire has been validated for the age group of 12–14. Although children were asked to provide their own opinions on the questions, guardians were asked to help in filling out the questionnaire. Whether this is a limitation or a strength can be debated, it certainly reflects the reality that many of the younger children would not be able to complete the questionnaire alone. We did not assay the input of the guardians. The participants’ social environment, for example family history of orthodontics might impact the results and was not assessed. The orthodontists (A.A and H.A) who did the screening, were not calibrated. However, all the orthodontists in Finland have been trained to use 10-grade scale. The findings and results of this study only represent the situation in one health center and the results are not generalizable.

## Conclusions

The main expectations for orthodontic treatment were to straighten the teeth, to get a better smile and to avoid problems in the future.

The self-perceived orthodontic treatment need of patients and their guardians was greater than treatment need assessed by the orthodontist.

To ensure optimal treatment, providing thorough information and engaging in discussions with both the individual and the guardian regarding the treatment need, anticipated treatment modality, treatment duration, and appointment frequency before commencing orthodontic treatment is important.

## Supplementary Material



## Data Availability

The data that support the findings of this study are available on request from the corresponding author (H.A).
