# Supplementary material for: Expectations of orthodontic treatment among 7–12 year-old children – a cross sectional study
Source: Acta Odontol Scand. 2025 Jun 18;84:43910. doi: 10.2340/aos.v84.43910 (PMC13063825; doi:10.2340/aos.v84.43910)
Supplement: Supplementary file 1 [file AOS-84-43910-s1.pdf]

Supplementary material has been published as submitted. It has not been copyedited or typeset by Acta Odontologica Scandinavica.

Questionnaire

Patients' expectations of orthodontic treatment process and results

Child's name:

Social security number :

Instructions: A parent/guardian fills in the questionnaire together with the child. Please read every question and answer by placing a cross (**X**) on the line at the most suitable place or by crossing the most suitable answer. **All the questions are concerning the child coming to the orthodontic screening appointment.** All the information is confidential and the answers don't have an effect on patient selection for orthodontic treatment. It takes about 5-10 minutes to answer the questionnaire.

NOTE. If you answer NO to the question number 1, so you feel that there is NO NEED for orthodontic treatment, you should not answer to other questions. Otherwise you should answer to all questions so that the information can be used for research purposes.

1) Do you feel that you (/ your child) have a need for orthodontic treatment?

YES / NO / MAYBE

2) Has the child's parents/guardians or siblings had orthodontic treatment before?

YES / NO / MAYBE

3) What type of orthodontic treatment do you (/your child) expect?

a) Braces, don't know what type?

0 5 10

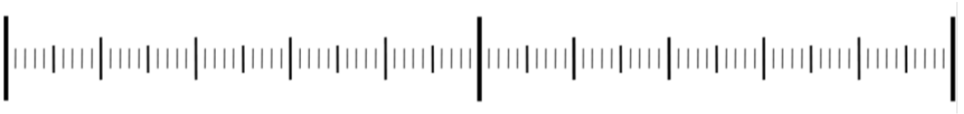

Extremely unlikely Extremely likely

b) Train track braces?

0 5 10

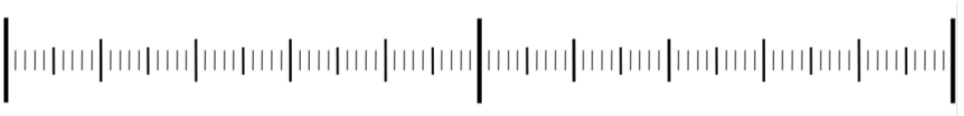

Extremely unlikely Extremely likely

c) Teeth removal?

0 5 10

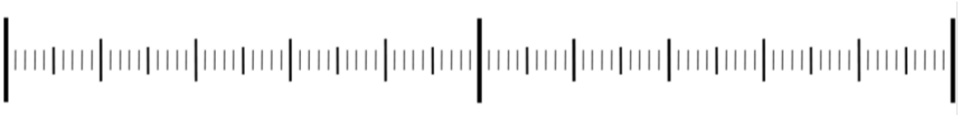

Extremely unlikely Extremely likely

d) Head brace or some other removable appliance placed into the mouth by yourself?

0 5 10

Extremely unlikely Extremely likely

e) Jaw surgery?

0 5 10

Extremely unlikely Extremely likely

4) Do you think orthodontic treatment will give you any problems?

0 5 10

Extremely unlikely Extremely likely

5) Do you think orthodontic treatment will be painful?

0 5 10

Extremely unlikely Extremely likely

6) Do you think orthodontic treatment will produce problems with eating?

0 5 10

Extremely unlikely Extremely likely

7) Do you expect orthodontic treatment to restrict what you (/ your child) can eat or drink?

0 5 10

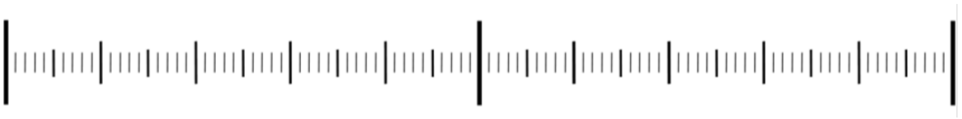

Extremely unlikely Extremely likely

8) How do you think people will react to you (/your child) wearing a brace?

0 5 10

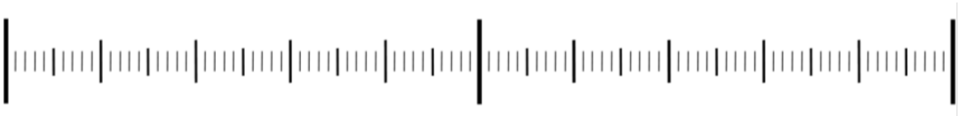

Negative reaction Positive reaction

9) How long do you expect orthodontic treatment to take?

4 years	
3,5 years	
3 years	
2,5 years	
2 years	
1,5 years	
1 year	
6 months	
3 months	
1 month	
Don't know	

10) How often do you think you will need to attend for check-up?

Every 8 months	
Every 6 months	
Every 3 months	
Every 2 months	
Every 6 weeks	
Every 4 weeks	
Every 2 weeks	
Once a week	
Twice a week	
Don't know	

11) Do you expect orthodontic treatment to:

a) Straighten your teeth?

0 5 10

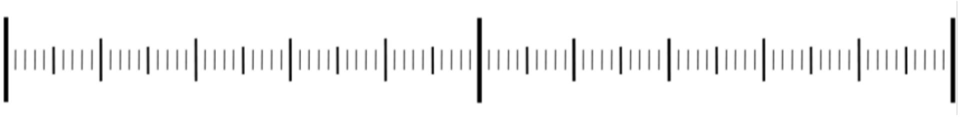

Extremely unlikely Extremely likely

b) Produce a better smile?

0 5 10

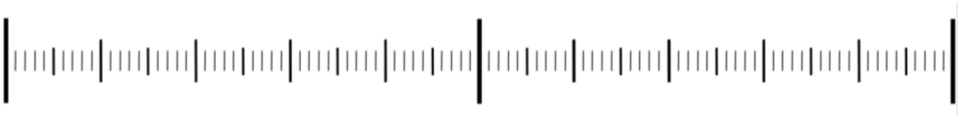

Extremely unlikely Extremely likely

c) Make it easier to eat?

0 5 10

Extremely unlikely Extremely likely

d) Make it easier to speak?

0 5 10

Extremely unlikely Extremely likely

e) Make it easier to keep my teeth clean?

0 5 10

Extremely unlikely Extremely likely

f) Improve my chances of a good career?

0 5 10

Extremely unlikely Extremely likely

g) Give you confidence socially?

0 5 10

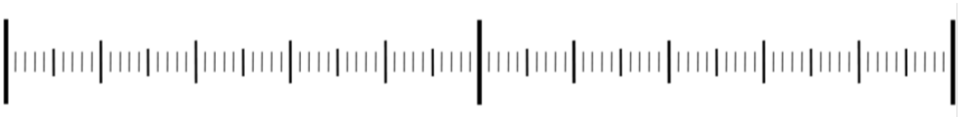

Extremely unlikely Extremely likely

h) Problems will be avoided in the future?

0 5 10

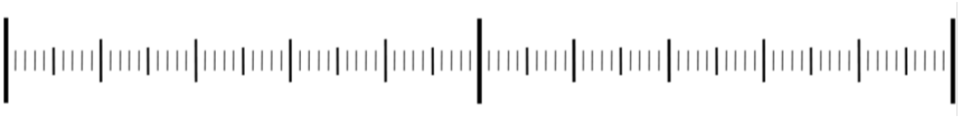

Extremely unlikely Extremely likely

Thank you for answering!
